# Organ allocation and transplant equity in Brazil: the hidden burden of HLA homozygosity and hypersensitization

**DOI:** 10.1590/2175-8239-JBN-2025-0294en

**Published:** 2026-05-18

**Authors:** Augusto Cesar Soares dos Santos, Evaldo Nascimento, Aline Regina da Cruz, Bernardo Vilela Nascimento, Raquel Aparecida Fabreti-Oliveira

**Affiliations:** 1 Faculdade de Ciências Médicas de Minas Gerais, Belo Horizonte, MG, Brazil.; 2 Universidade Federal de Minas Gerais, Hospital das Clínicas Ebserh, Belo Horizonte, MG, Brazil.; 3 Faculdade de Saúde da Santa Casa de Belo Horizonte, Belo Horizonte, MG, Brazil.; 4 Laboratório Imunolab Transplantes, Belo Horizonte, Brazil.

**Keywords:** Kidney Transplantation, HLA Antigens, Immunologic Sensitization, Unrelated Donors, Waiting Lists

## Abstract

**Introduction:**

Kidney transplantation remains the most cost-effective treatment for end-stage renal disease (ESRD); however, persistent organ scarcity and immunological barriers contribute to prolonged waiting times and inequitable access. Human leukocyte antigen (HLA) sensitization and homozygosity represent major challenges inadequately addressed by previous allocation models.

**Objectives:**

This study aimed to assess the impact of HLA sensitization and HLA homozygosity on access to kidney transplantation in the regional sector of Minas Gerais.

**Methods:**

We conducted a cross-sectional analysis of deceased-donor kidney transplant recipients and active candidates listed in the Brazilian National Transplant System (SNT) from January 2010 to August 2024 in a regional allocation sector of Minas Gerais. We assessed the impact of HLA sensitization and HLA-DRB1 homozygosity on access to transplantation, contextualizing the findings within recent national regulatory updates.

**Results:**

Of 2,907 patients analyzed, 1,794 (61.7%) underwent transplantation and 1,113 (38.3%) remained active on the waiting list. Diabetes mellitus (HR 1.177; p = 0.030) and blood groups A (HR 1.206; p < 0.001) and AB (HR 1.419; p = 0.002) were associated with increased access to transplantation. Higher levels of sensitization and HLA-DRB1 homozygosity were the strongest predictors of prolonged waiting time. These findings are consistent with inequities targeted by the Brazilian SNT regulatory revisions implemented in September 2025.

**Conclusion:**

Our results support recent Brazilian allocation reforms aimed at prioritizing hypersensitized and immunogenetically disadvantaged patients. Incorporating both the degree of sensitization and HLA-DR homozygosity into allocation algorithms represents a critical step toward improving equity in deceased-donor kidney transplantation.

## Introduction

Chronic kidney disease (CKD) is a major contributor to the global burden of noncommunicable diseases, affecting an estimated 850 million people worldwide and imposing substantial morbidity, mortality, and healthcare costs^1,2,3^. The burden is disproportionately concentrated in low- and lower-middle-income countries (LICs and LMICs), where access to early diagnosis, preventive strategies, and renal replacement therapies, including dialysis and transplantation, is limited^4,5^. Kidney transplantation is the most cost-effective treatment for end-stage renal disease (ESRD) and offers superior survival and quality of life compared with dialysis^6^. However, organ shortage results in prolonged waiting times and inequitable access^7^. Immunological barriers, particularly human leukocyte antigen (HLA) incompatibility and sensitization, further restrict access to transplantation^8^.

In Brazil, the National Transplant System (*Sistema Nacional de Transplantes*, SNT), coordinated by the Ministry of Health through the Unified Health System (*Sistema Único de Saúde*, SUS), operates the world’s largest public transplantation program^9^. Despite this comprehensive infrastructure, the kidney transplant waiting list remains long, with more than 41,900 patients registered, including over 32,900 actively waiting for a deceased-donor kidney^10,11^.

Historically, kidney allocation in Brazil relied on limited HLA typing (HLA-A, -B, and -DRB1), constraining donor–recipient matching and dis­advantaging sensitized candidates. This framework was updated in June 2025 with the expansion of deceased-donor HLA typing to include HLA-A, -B, -C, -DRB1/3/4/5, -DQA1, -DQB1, -DPA1, and -DPB1. This expansion enabled a more comprehensive and accurate identification of donor-specific antibodies (DSA) and greater reliability of virtual crossmatch analyses^12^. Additionally, while national policies initially did not account for HLA homozygosity, the state of São Paulo implemented a regional prioritization policy for HLA-DR–homozygous candidates in 2022^13^. These advances culminated in a nationwide revision of the kidney allocation guidelines in September 2025, which incorporated expanded HLA typing and standardized a recipient homozygosity scoring system based on HLA-DRB1 and HLA-DQB1 identities^14^.

This study aimed to assess the impact of HLA sensitization and HLA homozygosity on access to kidney transplantation in the regional sector of Minas Gerais prior to the most recent allocation policy changes. By analyzing how these immunological and genetic factors influence transplant likelihood, the study seeks to inform policy refinements that promote more equitable allocation practices and fairer access to transplantation for all candidates.

## Methods

### Study Design and Participants

This cross-sectional study evaluated kidney transplant recipients from deceased donors and active candidates registered in the Brazilian SNT between January 2010 and August 2024 within the regional sector of Minas Gerais state, Brazil. Kidney transplants were performed at multiple transplant centers located in Belo Horizonte, Minas Gerais. Data were obtained from the SNT computerized system and were restricted to patients whose pre-transplant immunological evaluations, including histocompatibility testing, were conducted by a single certified reference laboratory. Demographic, clinical, and immunological data were retrieved from the records of the Imunolab Transplant Laboratory, which is the official local reference laboratory for the SNT in this sector.

The study population comprised patients with end-stage kidney disease who met one of the following conditions during the study period: (i) receipt of a deceased-donor kidney transplant or (ii) active status on the kidney transplant waiting list at the time of data collection. Patients who were removed from the waiting list due to death, prolonged suspension, clinical ineligibility, administrative removal, treatment abandonment, recovery of renal function, or a personal decision not to pursue transplantation were excluded from the analytical cohort.

Given that the Brazilian kidney transplant waiting list is dynamic, with continuous entries and exits over time, the study population represents a selected subset of all listed candidates, restricted to individuals who either underwent transplantation or remained clinically eligible and active at the time of analysis. The study was approved by the Ethics Committee of the Faculty of Medical Sciences of Minas Gerais, Brazil (approval No. 2.122.409).

### Variables

Demographic variables included sex and age, while clinical variables encompassed the underlying kidney disease and the degree of sensitization, assessed by calculated panel-reactive antibody (cPRA), which reflects the likelihood of encountering DSA. Immunogenetic data included ABO blood type and HLA typing at the HLA-A, HLA-B, and HLA-DRB1 *loci*. Waiting time on the transplant list was calculated in months. All information was obtained from medical records and the SNT’s electronic database.

### Immunogenetic Evaluation

HLA typing was performed at intermediate resolution using sequence-specific oligonucleotide probes (LABType SSO kits, One Lambda). Homozygosity was defined by identical serologic antigens on both alleles at a given *locus*. Anti-HLA antibodies were detected using single antigen bead assays, and cPRA was calculated based on unacceptable antigens across class I and II *loci*. The cPRA value immediately preceding transplantation was used. Crossmatching was performed using complement-dependent cytotoxicity assays, and all transplanted patients had negative results.

### Statistical Analysis

Qualitative variables were described using absolute and relative frequencies, while quantitative variables were summarized by minimum, maximum, mean, standard deviation (SD), median, and interquartile range (first quartile [Q1] and third quartile [Q3]). To identify factors most strongly associated with the likelihood of transplantation, a decision tree model was constructed using the following variables: sex, age group, presence of diabetes mellitus, cPRA, blood type, and homozygosity at the HLA-A, HLA-B, and HLA-DR *loci*. The final model retained only the variables with the strongest association with the outcome, from which a cutoff point for the degree of sensitization was derived. Model performance was evaluated based on accuracy.

Transplantation risk was further examined through Kaplan–Meier survival analyses for cate­gorical variables, with median time to transplanta­tion (in months) and corresponding 95% confidence intervals (CIs) estimated. Univariate Cox proportional hazards models were fitted for each covariate. Variables with a p-value < 0.20 in univariate analysis were included in a multivariate Cox model, in which non-significant variables were sequentially excluded to arrive at a final model including only significant predictors. Sex was retained as a control variable regardless of its statistical significance. Results were reported as hazard ratios (HRs) with 95% CIs, and the proportional hazards assumption was tested using Schoenfeld residuals.

Allele proportions were estimated separately for each group, with simultaneous confidence intervals adjusted using Goodman’s method, appropriate for multinomial distributions. Differences between groups were considered statistically significant when the CI for the difference did not include zero. All statistical analyses were conducted in RStudio (version 2024.04.2) using R language (version 4.5.0). Statistical significance was set at p < 0.05.

## Results

Between January 2010 and August 2024, a total of 17,035 patients were registered on the kidney transplant waiting list in the state of Minas Gerais. During this period, 15,331 patients were removed from the list, including 7,814 who underwent kidney transplantation (from living or deceased donors) and 2,562 who died before transplantation. In the present study, 2,907 patients were included, comprising 1,794 (61.7%) individuals who underwent kidney transplantation and 1,113 (38.3%) who remained active candidates on the waiting list. These groups represent a subset of the overall state waiting list and were selected according to predefined eligibility criteria aligned with the objectives of this cross-sectional analysis.

Among those transplanted, 38 were prioritized due to contraindications to dialysis. The cohort was predominantly male (60.4%), with 76.9% of patients aged under 60 years (mean age: 49.3 years; SD 12.5). The most commonly reported underlying conditions were systemic arterial hypertension (19.1%) and diabetes mellitus (12.1%), although comorbidity data were missing or unreported in 41.2% of cases. Regarding blood type distribution, 48.8% of patients were identified as blood group O and 34.2% as group A. Homozygosity rates were 10.9% for HLA-A, 4.6% for HLA-B, and 8.9% for HLA-DR. The median cPRA value, used to assess immunological sensitization, was 0 [Q1 0; Q3 11], with 7.7% of patients exhibiting a cPRA ≥84. The median waiting time on the transplant list was 22.0 months [IQR 8.0–48.0]. Table 1 summarizes the distribution of characteristics according to transplant status (active on the list vs. transplanted), along with the median time to transplantation and hazard ratios (HRs) for transplant likelihood.

**Table 1:** Demographic and clinical characteristics of patients according to status

Features	Active on the list (n = 1,113)	Transplanted (n = 1,794)	Median time on the waiting list (months; 95% CI)	HR (95% CI)	P-value
**Demographic**					
Sex					
F	452 (39.3%)	699 (60.7%)	42 (38; 46)	–	–
M	661 (37.6%)	1,095 (62.4%)	40 (37; 44)	1.009 (0.917; 1.109)	0.859
Age, years				0.992 (0.988; 0.996)	**<0.001**
Minimum/Maximum	19.0/75.0	18.0/84.0			
Median [Q1; Q3]	50.0 [42.0; 59.0]	51.0 [41.0; 59.0]			
Mean (SD)	49.4 (12.1)	49.3 (12.8)			
Age group					
<60 years old	857 (38.3%)	1,379 (61.7%)	39 (36; 42)	1.000	–
≥60 years old	256 (38.2%)	415 (61.8%)	44 (41; 52)	0.906 (0.812; 1.012)	0.080
**Clinical**					
Underlying disease					
Glomerulonephritis	134 (53.0%)	119 (47.0%)	43 (36; 55)	1.000	–
Unknown	406 (33.9%)	792 (66.1%)	35 (31; 38)	1.208 (0.996; 1.465)	0.055
Diabetes mellitus	152 (43.2%)	200 (56.8%)	38 (30; 44)	1.247 (0.993; 1.564)	0.057
Polycystic kidney	60 (39.2%)	93 (60.8%)	42 (36; 64)	1.038 (0.791; 1.362)	0.786
Hypertension	196 (35.3%)	359 (64.7%)	49 (43; 54)	0.927 (0.753; 1.142)	0.476
Nephritis	42 (31.8%)	90 (68.2%)	48 (38; 68)	0.833 (0.633; 1.097)	0.193
Other	123 (46.6%)	141 (53.4%)	44 (32; 54)	0.943 (0.738; 1.205)	0.639
Diabetes mellitus					
No	961 (37.6%)	1,594 (62.4%)	42 (38; 44)	1.000	–
Yes	152 (43.2%)	200 (56.8%)	38 (30; 44)	1.177 (1.015; 1.364)	**0.030**
Blood type					
O	594 (41.8%)	826 (58.2%)	45 (42; 48)	1.000	–
A	333 (33.5%)	662 (66.5%)	36 (33; 41)	1.206 (1.089; 1.336)	**<0.001**
AB	46 (35.7%)	83 (64.3%)	36 (20; 43)	1.419 (1.132; 1.778)	**0.002**
B	140 (38.6%)	223 (61.4%)	40 (32; 46)	1.139 (0.983; 1.321)	0.084
cPRA		0.992 (0.990; 0.994)	**<0.001**		
Minimum/Maximum	0/100.0	0/100.0			
Median [Q1; Q3]	0 [0; 41.0]	0 [0; 5.0]			
Mean (SD)	22.1 (36.1)	11.2 (23.8)			
Categorized cPRA					
<84	949 (35.4%)	1,735 (64.6%)	38 (36; 41)	1.000	–
≥84	164 (73.5%)	59 (26.5%)	96 (84; 123)	0.368 (0.284; 0.476)	**<0.001**
HLA-A homozygotes					
No	966 (37.3%)	1,624 (62.7%)	41 (38; 43)	1.000	–
Yes	147 (46.4%)	170 (53.6%)	45 (35; 62)	0.872 (0.744; 1.021)	0.089
HLA-B homozygotes					
No	1,059 (38.2%)	1,713 (61.8%)	41 (38; 43)	1.000	–
Yes	54 (40.0%)	81 (60.0%)	51 (32; 69)	0.839 (0.671; 1.049)	0.124
HLA-DR homozygotes					
No	960 (36.2%)	1,689 (63.8%)	39 (39; 42)	1.000	–
Yes	153 (59.3%)	105 (40.7%)	73 (59; 104)	0.535 (0.439; 0.652)	**<0.001**

Abbreviations – HR: hazard ratio (calculated using a crude Cox model); CI: confidence interval; Q1: first quartile; Q3: third quartile; SD: standard deviation. Notes – Age refers to the age at the time of kidney transplantation for transplant recipients and to the age at the end of the study period for candidates who remained active on the waiting list.

Degree of sensitization and HLA-DR homozygosity were identified as the most important determinants of increased waiting time on the transplant waiting list. Patients with a cPRA ≥84 had an 8% chance of remaining active on the list. Likewise, patients with a cPRA <84 and HLA-DR homozygosity also had an 8% probability of remaining on the waiting list. In contrast, patients with a cPRA <84 and without HLA-DR homozygosity had an 84% likelihood of having received a transplant. The model demonstrated an accuracy of 66.36% (95% CI 64.61%–68.07%), indicating moderate discrimi­natory power. Therefore, a cutoff value of 84 for cPRA was used for subsequent analyses.

**Figure 1:**
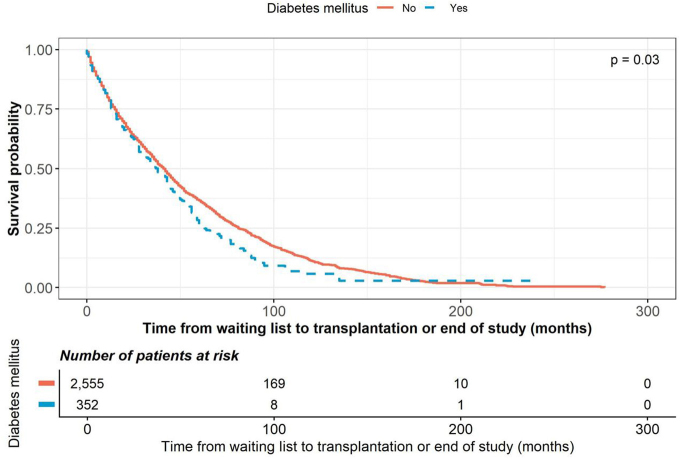
Kaplan–Meier curves showing the time from waiting list to transplantation, stratified by diabetes mellitus status.

**Figure 2:**
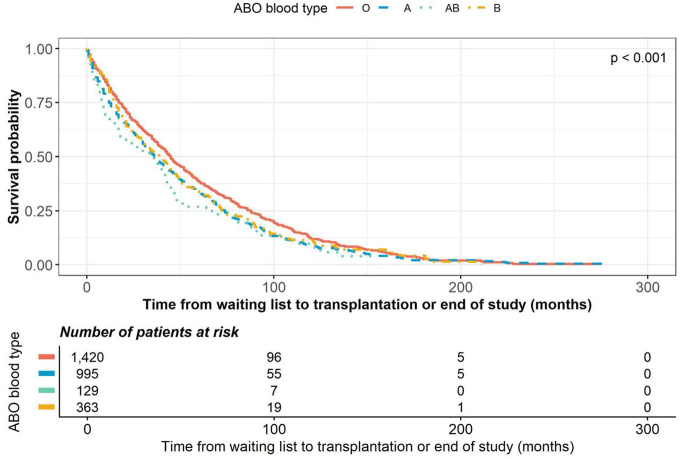
Kaplan–Meier curves showing the time from waiting list to transplantation, stratified by blood type.

**Figure 3:**
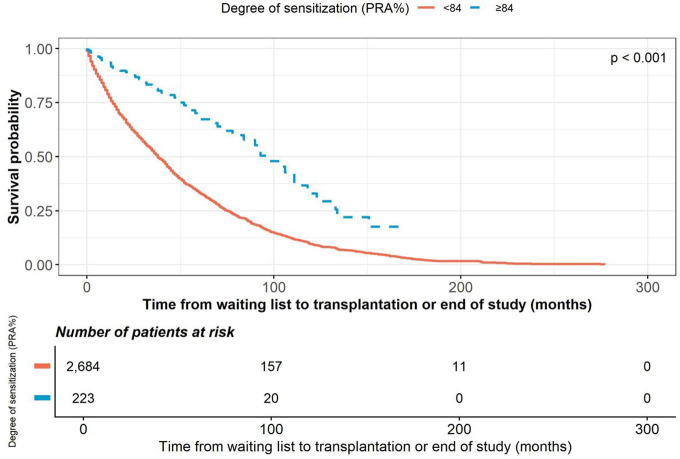
Kaplan–Meier curves showing the time from waiting list to transplantation, stratified by degree of sensitization.

**Figure 4:**
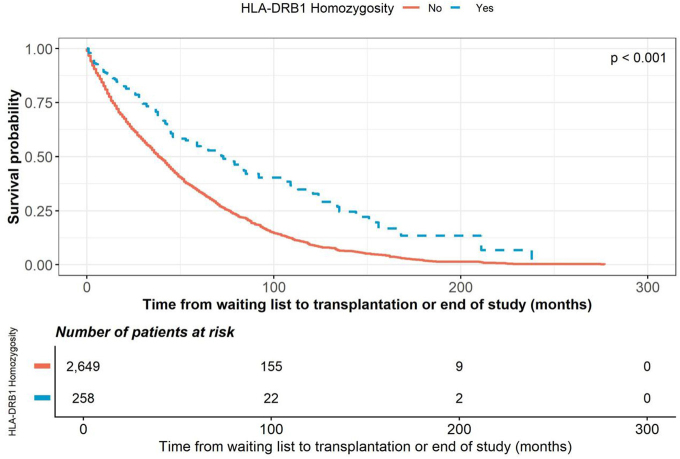
Kaplan–Meier curves showing the time from waiting list to transplantation, stratified by HLA-DR homozygosity status.

Factors associated with an increased likelihood of receiving a transplant included diabetes mellitus as the underlying disease (HR 1.177; p = 0.030) (Figure 1) and blood type A (HR 1.206; p < 0.001) or AB (HR 1.419; p = 0.002), compared with blood type O (Figure 2). In contrast, a lower probability of transplantation was observed among older individuals (HR 0.992; p < 0.001), those with a cPRA level ≥84 (HR 0.368; p < 0.001) (Figure 3), and patients with HLA-DR homozygosity (HR 0.535; p < 0.001) (Figure 4). No significant association was found between HLA-A or HLA-B homozygosity and transplant likelihood.

In the multivariate analysis (Table 2), several factors were linked to a higher likelihood of receiving a kidney transplant. These included being under 60 years old (HR 1.162; p = 0.009), having diabetes mellitus (HR 1.166; p = 0.046), and blood types A (HR 1.230; p < 0.001), AB (HR 1.447; p = 0.001), or B (HR 1.162; p = 0.047), when compared with blood type O. A lower cPRA value (<84) (HR 2.718; p < 0.001) and the absence of HLA-DR homozygosity (HR 1.824; p < 0.001) were also significantly associated with an increased probability of transplantation. According to the model, the profile with the highest predicted likelihood of receiving a transplant (HR 9.72) was that of a female under 60 years old, with diabetes, blood type AB, a cPRA value <84, and no HLA-DR homozygosity. In contrast, the individual with the lowest predicted probability (HR 0.95) was a male aged 60 or older, non-diabetic, with blood type O, a cPRA ≥84, and HLA-DR homozygosity.

**Table 2:** Adjusted Cox model for evaluation of risk factors for transplantation

Features	HR	95% CI	P-value
Sex			
F	1.000	–	–
M	0.952	(0.865; 1.048)	0.314
Age group			
<60 years old	1.162	(1.039; 1.300)	**0.009**
≥60 years old	1.000	–	–
Diabetes mellitus			
No	1.000	–	–
Yes	1.166	(1.003; 1.356)	**0.046**
Blood type			
O	1.000	–	–
A	1.230	(1.110; 1.363)	**<0.001**
AB	1.447	(1.154; 1.815)	**0.001**
B	1.162	(1.002; 1.348)	**0.047**
Categorized immune status			
<84	2.718	(2.093; 3.529)	**<0.001**
≥84	1.000	–	–
HLA-DR homozygotes			
No	1.824	(1.497; 2.222)	**<0.001**
Yes	1.000	–	–

Abbreviations – CI: confidence interval.

No significant association was observed between HLA-A and HLA-B allele distribution and transplantation status. However, the HLA-DR3 and HLA-DR8 alleles were less common among transplant recipients (Table 3).

**Table 3:** Frequency of HLA-A, HLA-B, and HLA-DR alleles according to transplant status

Characteristics	Active on the list (n = 2,226)	Transplanted (n = 3,588)	Differences (95% CI)
HLA-A			
A1	164 (7.4%)	276 (7.7%)	-0.3% (-2.5%; 1.8%)
A2	542 (24.3%)	930 (25.9%)	-1.6% (-5.1%; 2.0%)
A3	192 (8.6%)	302 (8.4%)	0.2% (-2.1%; 2.5%)
A9	0 (-)	1 (0.03%)	-0.03% (-0.1%; 0.06%)
A11	110 (4.9%)	180 (5.0%)	0.1% (-1.9%; 1.7%)
A23	151 (6.8%)	245 (6.8%)	0.04% (-2.1%; 2.0%)
A24	156 (7.0%)	290 (8.1%)	-1.1% (-3.2%; 1.1%)
A25	19 (0.9%)	34 (0.9%)	-0.1% (-0.9%; 0.7%)
A26	67 (3.0%)	71 (2.0%)	1.0% (-0.3%; 2.3%)
A29	115 (5.2%)	166 (4.6%)	0.5% (-1.3%; 2.3%)
A30	193 (8.7%)	306 (8.5%)	0.1% (-2.2%; 2.5%)
A31	86 (3.9%)	106 (3.0%)	0.9% (-0.6%; 2.4%)
A32	65 (2.9%)	102 (2.8%)	0.1% (-1.3%; 1.5%)
A33	64 (2.9%)	134 (3.7%)	-0.9% (-2.3%; 0.6%)
A34	31 (1.4%)	56 (1.6%)	-0.2% (-1.2%; 0.8%)
A36	25 (1.1%)	30 (0.8%)	0.3% (-0.5%; 1.1%)
A43	1 (0.04%)	0 (-)	0.04% (-0.1%; 0.2%)
A66	32 (1.4%)	52 (1.4%)	0.01% (-1.0%; 1.0%)
A68	163 (7.3%)	206 (5.7%)	1.6% (-0.5%; 3.6%)
A69	4 (0.2%)	5 (0.1%)	0.04% (-0.3%; 0.4%)
A74	39 (1.8%)	85 (2.4%)	-0.6% (-1.8%; 0.5%)
A80	7 (0.3%)	11 (0.3%)	0.01% (-0.5%; 0.5%)
HLA-B			
B5	0 (-)	3 (0.1%)	-0.1% (-0.2%; 0.1%)
B7	151 (6.8%)	254 (7.1%)	-0.3% (-2.5%; 1.9%)
B8	102 (4.6%)	157 (4.4%)	0.2% (-1.6%; 2.0%)
B13	37 (1.7%)	40 (1.1%)	0.5% (-0.5%; 1.6%)
B14	9 (0.4%)	17 (0.5%)	-0.1% (-0.6%; 0.5%)
B15	28 (1.3%)	30 (0.8%)	0.4% (-0.5%; 1.3%)
B17	0 (-)	1 (0.03%)	-0.03% (-0.1%; 0.1%)
B18	111 (5.0%)	158 (4.4%)	0.6% (-1.3%; 2.5%)
B21	0 (-)	1 (0.03%)	-0.03% (-0.1%; 0.1%)
B27	62 (2.8%)	71 (2.0%)	0.8% (-0.6%; 2.2%)
B35	204 (9.2%)	375 (10.5%)	-1.3% (-3.9%; 1.3%)
B37	30 (1.3%)	32 (0.9%)	0.5% (-0.5%; 1.4%)
B38	25 (1.1%)	48 (1.3%)	-0.2% (-1.2%; 0.7%)
B39	67 (3.0%)	79 (2.2%)	0.8% (-0.6%; 2.2%)
B40	15 (0.7%)	9 (0.3%)	0.4% (-0.2%; 1.1%)
B41	26 (1.2%)	39 (1.1%)	0.1% (-0.9%; 1.0%)
B42	69 (3.1%)	92 (2.6%)	0.5% (-0.9%; 2.0%)
B44	214 (9.6%)	425 (11.8%)	-2.2% (-4.9%; 0.5%)
B45	69 (3.1%)	128 (3.6%)	-0.5% (-2.0%; 1.1%)
B46	1 (0.04%)	0 (-)	0.04% (-0.1%; 0.2%)
B47	4 (0.2%)	2 (0.1%)	0.1% (-0.2%; 0.4%)
B48	14 (0.6%)	11 (0.3%)	0.3% (-0.3%; 0.9%)
B49	72 (3.2%)	103 (2.9%)	0.4% (-1.2%; 1.9%)
B50	48 (2.2%)	67 (1.9%)	0.3% (-1.0%; 1.5%)
B51	152 (6.8%)	268 (7.5%)	-0.6% (-2.9%; 1.6%)
B52	51 (2.3%)	91 (2.5%)	-0.2% (-1.6%; 1.1%)
B53	111 (5.0%)	135 (3.8%)	1.2% (-0.6%; 3.1%)
B54	0 (-)	2 (0.1%)	-0.1% (-0.2%; 0.1%)
B55	21 (0.9%)	25 (0.7%)	0.2% (-0.6%; 1.1%)
B56	2 (0.1%)	9 (0.3%)	-0.2% (-0.5%; 0.2%)
B57	55 (2.5%)	110 (3.1%)	-0.6% (-2.0%; 0.8%)
B58	66 (3.0%)	115 (3.2%)	-0.2% (-1.8%; 1.3%)
B60	42 (1.9%)	63 (1.8%)	0.1% (-1.1%; 1.3%)
B61	37 (1.7%)	64 (1.8%)	-0.1% (-1.3%; 1.0%)
B62	69 (3.1%)	113 (3.1%)	-0.04% (-1.6%; 1.5%)
B63	27 (1.2%)	53 (1.5%)	-0.3% (-1.3%; 0.7%)
B64	19 (0.9%)	38 (1.1%)	-0.2% (-1.1%; 0.6%)
B65	83 (3.7%)	133 (3.7%)	0.02% (-1.7%; 1.7%)
B70	3 (0.1%)	9 (0.3%)	-0.1% (-0.5%; 0.3%)
B71	44 (2.0%)	74 (2.1%)	-0.1% (-1.3%; 1.2%)
B72	54 (2.4%)	106 (3.0%)	-0.5% (-1.9%; 0.9%)
B73	5 (0.2%)	2 (0.1%)	0.2% (-0.2%; 0.5%)
B75	9 (0.4%)	6 (0.2%)	0.2% (-0.3%; 0.7%)
B78	1 (0.04%)	5 (0.1%)	-0.1% (-0.3%; 0.2%)
B81	15 (0.7%)	22 (0.6%)	0.1% (-0.6%; 0.8%)
B82	2 (0.1%)	3 (0.1%)	0.01% (-0.3%; 0.3%)
HLA-DR			
DR1	232 (10.4%)	333 (9.3%)	1.1% (-1.2%; 3.5%)
DR3	29 (1.3%)	16 (0.4%)	**0.9% (0.1%; 1.6%)**
DR4	269 (12.1%)	422 (11.8%)	0.3% (-2.3%; 2.9%)
DR7	269 (12.1%)	453 (12.6%)	-0.5% (-3.1%; 2.1%)
DR8	170 (7.6%)	202 (5.6%)	**2.0% (0.1%; 4.1%)**
DR9	49 (2.2%)	57 (1.6%)	0.6% (-0.5%; 1.7%)
DR10	58 (2.6%)	82 (2.3%)	0.3% (-0.9%; 1.6%)
DR11	241 (10.8%)	440 (12.3%)	-1.4% (-4.0%; 1.1%)
DR12	37 (1.7%)	77 (2.1%)	-0.5% (-1.6%; 0.6%)
DR13	290 (13.0%)	512 (14.3%)	-1.2% (-4.0%; 1.5%)
DR14	62 (2.8%)	106 (3.0%)	-0.2% (-1.5%; 1.2%)
DR15	211 (9.5%)	371 (10.3%)	-0.9% (-3.2%; 1.5%)
DR16	63 (2.8%)	114 (3.2%)	-0.3% (-1.7%; 1.0%)
DR17	184 (8.3%)	334 (9.3%)	-1.0% (-3.3%; 1.2%)
DR18	62 (2.8%)	69 (1.9%)	0.9% (-0.4%; 2.1%)

Abbreviations – CI: confidence interval, adjusted using Goodman’s method.

## Discussion

This study evaluated the factors associated with deceased-donor kidney transplantation and waiting time in a regional cohort from Minas Gerais prior to the nationwide revision of the Brazilian kidney allocation guidelines in September 2025, which incorporated expanded HLA typing and standardized a recipient homozygosity scoring system based on HLA-DRB1 and HLA-DQB1 identities. Our findings highlight the central role of immunological status, particularly HLA homozygosity and sensitization, as well as demographic and clinical factors, including age, diabetes mellitus, and blood type, in determining transplantation likelihood.

Diabetes mellitus was associated with a higher probability of kidney transplantation in this cohort. This finding should be interpreted in the context of the Brazilian allocation policy, in which diabetes mellitus (type 1 or type 2) is a clinical criterion that confers additional priority points. Consequently, diabetic candidates may be transplanted earlier when compatible organs become available, while those remaining active on the waiting list represent a selected survivor cohort with lower immediate clinical risk. Increasing age was associated with a reduced likelihood of transplantation, likely reflecting greater comorbidity burden, poorer expected post-transplant outcomes, and preferential allocation of deceased-donor kidneys to younger candidates. As expected, blood type also influenced access, with blood groups A and AB demonstrating higher trans­plantation likelihood compared with blood group O.

Our predictive model demonstrated that HLA-DR homozygosity and cPRA are key determinants of reduced access to kidney transplantation. Homozygosity at the HLA-DR *locus* was associated with a lower likelihood of transplantation, consistent with previous studies reporting prolonged waiting times among homozygous candidates^15,16,17^. Homozygous recipients may be disadvantaged under allocation systems that prioritize HLA compatibility without adequately accounting for the immunogenetic constraints imposed by homozygosity. This phenomenon has been previously described by De Marco et al.^16^, who reported a significantly higher frequency of HLA-DRB1 homozygosity among transplant candidates compared with the general population.

Highly sensitized patients, characterized by elevated cPRA values, face substantial barriers to transplantation due to broader alloimmune reactivity and reduced availability of compatible donors^15,16,17,18,19^. In our cohort, candidates with cPRA ≥84 had a markedly lower likelihood of transplantation, reinforcing the role of sensitization as a major determinant of prolonged waiting time. The interplay between HLA homozygosity and sensitization further compounds immunological disadvantage, as homozygosity may contribute to the development or persistence of high levels of anti-HLA antibodies.

This study has several limitations. Although all eligible patients during the study period were included, no formal sample size calculation was performed. Selection bias was partially mitigated by restricting analyses to individuals evaluated by a single reference laboratory; however, the retrospective design introduces the potential for residual confounding and information bias. In addition, comorbidity data were missing or incomplete in a substantial proportion of cases, underscoring the need to improve data completeness and standardization within the Brazilian National Transplant System (SNT) registry. The analysis of HLA allele frequencies should be interpreted cautiously. To address the issue of multiple comparisons, allele proportions were estimated separately for each group, with simultaneous confidence intervals adjusted according to Goodman’s method, appropriate for multinomial distributions. No formal hypothesis testing across multiple alleles was performed, and the HLA frequency analysis was therefore considered exploratory. Observed differences between groups were interpreted conservatively and were considered meaningful only when the CI for the difference did not include zero.

## Conclusion

As the global burden of chronic kidney disease continues to rise, particularly in low- and middle-income countries, optimizing kidney allocation policies remains essential to improve equity and access to transplantation. This study demonstrates that HLA-DRB1 homozygosity and hypersensitization are associated with reduced access to deceased-donor kidney transplantation and longer waiting times in Brazil.

Recent revisions to the Brazilian kidney allocation policy, including the classification of donor–recipient pairs as HLA-DRB1 identical when both are homozygous for the same DRB1 antigen and measures addressing sensitized candidates, represent important steps toward mitigating these disparities. Continued evaluation and refinement of allocation algorithms that account for both HLA homozygosity and sensitization are warranted to improve fairness and efficiency in kidney transplantation in Brazil.

## Data Availability

The data generated in this study may be made available upon request.

## References

[1] Calice-Silva V, Neyra JA, Ferreiro Fuentes A, Singer Wallbach Massai KK, Arruebo S, Bello AK (2024). Capacity for the management of kidney failure in the
International Society of Nephrology Latin America region: report from the
2023 ISN Global Kidney Health Atlas (ISN-GKHA). Kidney Int Suppl.

[2] Bikbov B, Purcell CA, Levey AS, Smith M, Abdoli A, Abebe M (2020). Global, regional, and national burden of chronic kidney disease,
1990–2017: a systematic analysis for the Global Burden of Disease Study
2017. Lancet.

[3] Vassalotti JA, Francis A, Soares dos Santos AC, Correa-Rotter R, Abdellatif D, Hsiao LL (2025). Are your kidneys Ok? Detect early to protect kidney
health. Kidney Int.

[4] Jager KJ, Kovesdy C, Langham R, Rosenberg M, Jha V, Zoccali C (2019). A single number for advocacy and communication: worldwide more
than 850 million individuals have kidney diseases. Nephrol Dial Transplant.

[5] Francis A, Harhay MN, Ong ACM, Tummalapalli SL, Ortiz A, Fogo AB (2024). Chronic kidney disease and the global public health agenda: an
international consensus. Nat Rev Nephrol.

[6] Abecassis M, Bartlett ST, Collins AJ, Davis CL, Delmonico FL, Friedewald JJ (2008). Kidney transplantation as primary therapy for end-stage renal
disease. Clin J Am Soc Nephrol.

[7] Cron DC, Patzer RE, Adler JT (2025). Supply, demand, and a growing US Kidney Transplant Waiting
List. JAMA Netw Open.

[8] Iyer HS, Jackson AM, Montgomery RA (2014). Sensitized patients, transplant, and management. Curr Transplant Rep.

[9] Foresto RD, Pestana JOM, Silva HT (2020). Brasil: the leading public kidney transplant program
worldwide. Rev Assoc Med Bras.

[10] Brasil, Ministério da Saúde (2024). Relatório de lista de espera por um transplante de órgão ou
córnea (Brasil) - Série histórica 2008-2023. Sistema Nacional de Transplantes.

[11] Brasil, Ministério da Saúde (2017). Consolidação das normas sobre os sistemas e os subsistemas do
Sistema Único de Saúde. Portaria de Consolidação n^o^ 04, de 28 de setembro de
2017.

[12] Brasil, Minsterio da Saude (2025). Exclui, inclui e altera procedimentos na Tabela de Procedimentos,
Medicamentos, Órteses, Próteses e Materiais Especiais do SUS (Tabela de
Procedimentos do SUS) e estabelece recurso a ser disponibilizado a Estados e
Municípios. Portaria GM/MS n^o^ 7.076, de 9 de junho de 2025.

[13] De Marco R, Monteiro F, Requião-Moura LR, Medina-Pestana J, Gerbase-Delima M (2023). The problem and the solution for equitable access of HLA-DR
homozygous patients to kidney transplantation. Transplantation.

[14] Brasil, Ministerio da Saude (2025). Altera a Portaria de Consolidação GM/MS nº 4 de 28 de setembro de
2017, para estabelecer a Política Nacional de Doação e Transplantes e
definir o Regulamento Técnico do Sistema Nacional de
Transplantes. Portaria GM/MS n^o^ 8.041, de 25 de setembro de 2025.

[15] Loeffler-Wirth H, Lehmann C, Lachmann N, Doxiadis I (2024). Homozygosity in any HLA locus is a risk factor for specific
antibody production: the taboo concept 2.0. Front Immunol.

[16] de Marco R, Monteiro F, Requião-Moura LR, Medina-Pestana J, Gerbase-DeLima M (2023). The problem and the solution for equitable access of HLA-DR
homozygous patients to kidney transplantation. Transplantation.

[17] Rushakoff JA, Gragert L, Pando MJ, Stewart D, Huang E, Kim I (2022). HLA homozygosity and likelihood of sensitization in kidney
transplant candidates. Transplant Direct.

[18] Lee JH, Koo TY, Lee JE, Oh KH, Kim BS, Yang J (2024). Impact of sensitization and ABO blood types on the opportunity of
deceased-donor kidney transplantation with prolonged waiting
time. Sci Rep.

[19] Sousa KMP, Barbosa NB, Araujo IFR, Freitas LC, Ponte MF, Silva PGB (2021). Análise da influência da homozigose dos alelos HLA no tempo de
espera para realização do transplante renal no Ceará. Res Soc Dev.

