# Supplementary material for: Organ allocation and transplant equity in Brazil: the hidden burden of HLA homozygosity and hypersensitization
Source: J Bras Nefrol. 2026 May 18;48(3):e20250294. doi: 10.1590/2175-8239-JBN-2025-0294en (PMC13183359; doi:10.1590/2175-8239-JBN-2025-0294en)
Supplement: Translation

# Translation: Alocação de órgãos e equidade no transplante no Brasil: os desafios da homozigosidade e da hipersensibilização ao HLA

**DOI:** 10.1590/2175-8239-JBN-2025-0294pt

**Authors:** Augusto Cesar Soares dos Santos, Evaldo Nascimento, Aline Regina da Cruz, Bernardo Vilela Nascimento, Raquel Aparecida Fabreti-Oliveira

**Affiliations:** 1 Faculdade de Ciências Médicas de Minas Gerais, Belo Horizonte, MG, Brasil.; 2 Universidade Federal de Minas Gerais, Hospital das Clínicas Ebserh, Belo Horizonte, MG, Brasil.; 3 Faculdade de Saúde da Santa Casa de Belo Horizonte, Belo Horizonte, MG, Brasil.; 4 Laboratório Imunolab Transplantes, Belo Horizonte, Brasil.

**Keywords:** Transplante de Rim, Antígenos HLA, Sensibilização Imunológica, Doadores não Relacionados, Listas de Espera

## Abstract

**Introdução:**

O transplante renal permanece o tratamento mais custo-efetivo para doença renal em estágio terminal. A sensibilização aos antígenos leucocitários humanos (HLA) e a homozigosidade representam desafios relevantes que não foram adequadamente abordados pelos modelos de alocação anteriores.

**Objetivos:**

Avaliar o impacto da sensibilização ao HLA e da homozigosidade HLA no acesso ao transplante renal no setor regional de Minas Gerais.

**Métodos:**

Realizou-se uma análise transversal de receptores de transplante renal com doador falecido e de candidatos ativos listados no Sistema Nacional de Transplantes (SNT) entre janeiro de 2010 e agosto de 2024, no setor regional de Minas Gerais. Avaliou-se o impacto da sensibilização ao HLA e da homozigosidade HLA-DRB1 no acesso ao transplante.

**Resultados:**

Dos 2.907 pacientes analisados, 1.794 (61,7%) foram trans­plantados e 1.113 (38,3%) permaneceram ativos na lista de espera. Diabetes mellitus (HR 1,177; p = 0,030) e os grupos sanguíneos A (HR 1,206; p < 0,001) e AB (HR 1,419; p = 0,002) associaram-se a maior acesso ao transplante. Maior grau de sensibilização e homozigosidade HLA-DRB1 foram os principais preditores de tempo de espera prolongado. Esses achados são consistentes com as iniquidades abordadas pelas revisões regulatórias do SNT brasileiro implementadas em setembro de 2025.

**Conclusão:**

Nossos resultados sustentam as recentes reformas realizadas no sistema de alocação brasileiro voltadas à priorização de pacientes hipersensibilizados e imunogeneticamente desfavorecidos. A incorporação tanto do grau de sensibilização quanto da homozigosidade HLA-DR nos algoritmos de alocação representa um passo crítico para aprimorar a equidade no transplante renal com doador falecido.

## Introdução

A doença renal crônica (DRC) é um dos principais contribuintes para a carga global de doenças não transmissíveis, afetando aproximadamente 850 milhões de pessoas em todo o mundo e impondo significativa morbidade, mortalidade e custos aos sistemas de saúde^1,2,3^. A carga está despropor­cionalmente concentrada em países de renda baixa e média baixa, onde o acesso ao diagnóstico precoce, às estratégias de prevenção e às terapias renais substitutivas, incluindo diálise e transplante, é limitado^4,5^. O transplante renal é o tratamento mais custo-efetivo para a doença renal terminal (DRT) e oferece sobrevida e qualidade de vida superiores em comparação com a diálise^6^. No entanto, a escassez de órgãos leva a longos tempos de espera e a disparidades no acesso^7^. Barreiras imuno­lógicas, particularmente a incompatibilidade e a sensibilização aos antígenos leucocitários humanos (HLA), restringem ainda mais o acesso ao transplante^8^.

No Brasil, o Sistema Nacional de Transplantes (SNT), coordenado pelo Ministério da Saúde, gerencia o maior programa público de transplante de órgãos do mundo por meio do Sistema Único de Saúde (SUS)^9^. Apesar da extensa infraestrutura, a lista de espera para transplante renal continua longa, com mais de 41.900 pacientes registrados, incluindo mais de 32.900 aguardando ativamente por um rim de doador falecido^10,11^.

Historicamente, a alocação de rins no Brasil baseava-se em uma tipagem HLA limitada aos HLA-A, -B e -DRB1, o que restringia o pareamento entre doador e receptor e desfavorecia candidatos sen­sibilizados. Esse arcabouço foi atualizado em junho de 2025 com a ampliação da tipagem HLA de doadores falecidos para incluir HLA-A, -B, -C, -DRB1/ 3/4/5, -DQA1, -DQB1, -DPA1 e -DPB1, permitindo uma identificação mais abrangente e precisa de anticorpos específicos contra o doador (DSA) e maior confiabilidade das análises de *crossmatch* virtual^12^. Além disso, embora as políticas nacionais inicialmente não contemplassem a homozigosidade HLA, o estado de São Paulo implementou, em 2022, uma política regional de priorização para candidatos homozigotos para HLA-DR^13^. Esses avanços culminaram em uma revisão nacional das diretrizes de alocação de rins em setembro de 2025, que incorporou a tipagem HLA ampliada e padronizou um sistema de pontuação de homozigosidade do receptor baseado nas identidades HLA-DRB1 e HLA-DQB1^14^.

Este estudo teve como objetivo avaliar o impacto da sensibilização ao HLA e da homozigosidade HLA no acesso ao transplante renal no setor regional de Minas Gerais antes das recentes alterações regulatórias. Ao analisar como esses fatores imunológicos e genéticos influenciam a probabilidade de transplante, o estudo busca informar refinamentos nas políticas que promovam práticas de alocação mais equitativas e garantam um acesso mais justo ao transplante para todos os candidatos.

## Métodos

### Desenho do Estudo e Participantes

Este estudo transversal avaliou receptores de transplante renal com doador falecido e candidatos ativos registrados no Sistema Nacional de Transplantes (SNT) entre janeiro de 2010 e agosto de 2024, no setor regional do Estado de Minas Gerais, Brasil. Os transplantes renais foram realizados em múltiplos centros transplantadores localizados em Belo Horizonte, Minas Gerais. Os dados foram obtidos a partir do sistema informatizado do SNT e restringiram-se a pacientes cujas avaliações imunológicas pré-transplante, incluindo testes de histocompatibilidade, foram realizadas por um único laboratório de referência certificado. As informações demográficas, clínicas e imunológicas foram extraídas dos registros do Laboratório Imunolab Transplantes, o laboratório oficial de referência local do SNT nesse setor.

A população do estudo foi composta por pacientes com doença renal em estágio terminal que atenderam a um dos seguintes critérios durante o período do estudo: (i) realização de transplante renal com doador falecido ou (ii) *status* ativo na lista de espera para transplante renal no momento da coleta de dados. Foram excluídos da coorte analítica os pacientes removidos da lista de espera por óbito, suspensão prolongada, ineligibilidade clínica, exclusão administrativa, abandono do tratamento, recuperação da função renal ou decisão pessoal de não prosseguir com o transplante.

Como a lista de espera para transplante renal no Brasil é dinâmica, com entradas e saídas contínuas ao longo do tempo, a população do estudo representa um subconjunto selecionado de todos os candidatos listados, restrito a indivíduos transplantados ou que permaneceram clinicamente elegíveis e ativos no momento da análise. O estudo foi aprovado pelo Comitê de Ética da Faculdade de Ciências Médicas de Minas Gerais (parecer n° 2.122.409).

### Variáveis

As variáveis demográficas incluíram sexo e idade, enquanto as variáveis clínicas englobaram a doença renal de base e o grau de sensibilização, avaliado pelo nível de anticorpos reativos contra painel (cPRA), que reflete o risco de encontrar DSA. Os dados imunogenéticos incluíram tipagem sanguínea ABO e tipagem HLA nos *loci* HLA-A, HLA-B e HLA-DRB1, juntamente com a distribuição de variantes antigênicas nesses *loci*. O tempo de espera em lista foi calculado em meses. Todas as informações foram obtidas dos prontuários médicos e do banco de dados eletrônico do SNT.

### Avaliação Imunogenética

A tipagem HLA foi realizada em resolução inter­mediária por meio de sondas de oligonucleotídeos específicos de sequência (kits *LABType SSO*, *One Lambda*). A homozigosidade foi definida pela presença de antígenos sorológicos idênticos em ambos os alelos de um determinado *locus*. Os anticorpos anti-HLA foram detectados por ensaios de *beads* de antígeno único, e o cPRA foi calculado com base nos antígenos inaceitáveis nos *loci* das classes I e II. Utilizou-se o valor desse índice imediatamente anterior ao transplante. O *cross­match* foi realizado por ensaios de citotoxicidade dependente de complemento, e todos os pacientes transplantados apresentaram resultados negativos.

### Análise Estatística

As variáveis qualitativas foram descritas por meio de frequências absolutas e relativas, enquanto as variáveis quantitativas foram resumidas por mínimo, máximo, média, desvio padrão (DP), mediana e intervalo interquartil (primeiro quartil [Q1] e terceiro quartil [Q3]). Para identificar os fatores mais fortemente associados à probabilidade de transplante, foi construído um modelo de árvore de decisão utilizando as seguintes variáveis: sexo, faixa etária, presença de diabetes mellitus, cPRA, tipo sanguíneo e homozigosidade nos *loci* HLA-A, HLA-B e HLA-DR. O modelo final manteve apenas as variáveis com maior associação com o desfecho, a partir das quais foi derivado um ponto de corte para o grau de sensibilização. O desempenho do modelo foi avaliado com base na precisão.

O risco de transplante foi examinado por meio de análises de sobrevida de *Kaplan-Meier* para variáveis categóricas, com tempo médio até o transplante (em meses) e intervalos de confiança (ICs) de 95% correspondentes estimados. Modelos univariados de riscos proporcionais de *Cox* foram ajustados para cada covariável. As variáveis com valor de p < 0,20 na análise univariada foram incluídas em um modelo multivariado de Cox, no qual as variáveis não significativas foram excluídas sequencialmente para chegar a um modelo final, incluindo apenas preditores significativos. O sexo foi mantido como variável de controle, independentemente de sua significância estatística. Os resultados foram relatados como razões de risco (HRs) com ICs de 95%, e a suposição de riscos proporcionais foi testada utilizando resíduos de *Schoenfeld*.

As proporções alélicas foram estimadas separadamente para cada grupo, com intervalos de confiança simultâneos ajustados pelo método de *Goodman*, apropriado para distribuições multinomiais. As diferenças entre os grupos foram consideradas estatisticamente significativas quando o IC da diferença não incluía zero. Todas as análises estatísticas foram realizadas no *RStudio* (versão 2024.04.2), utilizando a linguagem R (versão 4.5.0). A significância estatística foi estabelecida em p < 0,05.

## Resultados

Entre janeiro de 2010 e agosto de 2024, um total de 17.035 pacientes foi registrado na lista de espera para transplante renal no estado de Minas Gerais. Durante esse período, 15.331 pacientes foram removidos da lista, incluindo 7.814 que realizaram transplante renal (com doador vivo ou falecido) e 2.562 que evoluíram a óbito antes do transplante. No presente estudo, foram incluídos 2.907 pacientes, sendo 1.794 (61,7%) indivíduos transplantados e 1.113 (38,3%) que permaneceram como candidatos ativos na lista de espera. Esses grupos representam um subconjunto da lista estadual total, tendo sido selecionados de acordo com critérios de elegibilidade previamente definidos, alinhados aos objetivos desta análise transversal.

Entre os transplantados, 38 foram priorizados devido a contraindicações à diálise. A coorte era predominantemente do sexo masculino (60,4%), com 76,9% dos pacientes com menos de 60 anos (média de idade: 49,3 anos; DP 12,5). As condições básicas mais comumente relatadas foram hipertensão arterial sistêmica (19,1%) e diabetes mellitus (12,1%), embora os dados de comorbidade estivessem ausentes ou não relatados em 41,2% dos casos. Em relação à distribuição do tipo sanguíneo, 48,8% dos pacientes foram identificados como grupo sanguíneo O e 34,2% como grupo A. As taxas de homozigosidade foram de 10,9% para HLA-A, 4,6% para HLA-B e 8,9% para HLA-DR. O valor mediano de cPRA, utilizado para avaliar a sensibilização imunológica, foi 0 [Q1 0; Q3 11], com 7,7% dos pacientes apresentando escore cPRA *≥*84. O tempo médio de espera na lista de transplantes foi de 22,0 meses [IIQ 8,0–48,0]. A Tabela 1 resume a distribuição das características de acordo com o *status* do transplante (ativo na lista vs. transplantado), juntamente com o tempo médio até o transplante e as razões de risco (HRs) para o risco de transplante.

**Tabela 1:** Características demográficas e clínicas dos pacientes de acordo com o *status*

Características	Ativo na lista (n = 1.113)	Transplantado (n = 1.794)	Tempo médio na lista de espera (meses; IC 95%)	HR (IC 95%)	Valor de p
**Demográficas**					
Sexo					
F	452 (39.3%)	699 (60,7%)	42 (38; 46)	–	–
M	661 (37.6%)	1,095 (62,4%)	40 (37; 44)	1,009 (0,917; 1,109)	0,859
Idade, anos				0,992 (0,988; 0,996)	**<0,001**
Mínimo/Máximo	19.0/75.0	18,0/84,0			
Mediana [Q1; Q3]	50.0 [42.0; 59.0]	51,0 [41,0; 59,0]			
Média (DP)	49.4 (12.1)	49,3 (12,8)			
Faixa etária					
<60 anos	857 (38.3%)	1,379 (61,7%)	39 (36; 42)	1,000	–
≥60 anos	256 (38.2%)	415 (61,8%)	44 (41; 52)	0,906 (0,812; 1,012)	0,080
**Clínicas**					
Doença subjacente					
Glomerulonefrite	134 (53.0%)	119 (47,0%)	43 (36; 55)	1,000	–
Desconhecida	406 (33.9%)	792 (66,1%)	35 (31; 38)	1,208 (0,996; 1,465)	0,055
Diabetes mellitus	152 (43.2%)	200 (56,8%)	38 (30; 44)	1,247 (0,993; 1,564)	0,057
Rim policístico	60 (39.2%)	93 (60,8%)	42 (36; 64)	1,038 (0,791; 1,362)	0,786
Hipertensão	196 (35.3%)	359 (64,7%)	49 (43; 54)	0,927 (0,753; 1,142)	0,476
Nefrite	42 (31.8%)	90 (68,2%)	48 (38; 68)	0,833 (0,633; 1,097)	0,193
Outro	123 (46.6%)	141 (53,4%)	44 (32; 54)	0,943 (0,738; 1,205)	0,639
Diabetes mellitus					
Não	961 (37.6%)	1,594 (62,4%)	42 (38; 44)	1,000	–
Sim	152 (43.2%)	200 (56,8%)	38 (30; 44)	1,177 (1,015; 1,364)	**0,030**
Tipo sanguíneo					
O	594 (41.8%)	826 (58,2%)	45 (42; 48)	1,000	–
A	333 (33.5%)	662 (66,5%)	36 (33; 41)	1,206 (1,089; 1,336)	**<0,001**
AB	46 (35.7%)	83 (64,3%)	36 (20; 43)	1,419 (1,132; 1,778)	**0,002**
B	140 (38.6%)	223 (61,4%)	40 (32; 46)	1,139 (0,983; 1,321)	0,084
cPRA		0,992 (0,990; 0,994)	**<0,001**		
Mínimo/Máximo	0/100.0	0/100,0			
Mediana [Q1; Q3]	0 [0; 41.0]	0 [0; 5,0]			
Média (DP)	22.1 (36.1)	11,2 (23,8)			
cPRA categorizado					
<84	949 (35.4%)	1,735 (64,6%)	38 (36; 41)	1,000	–
≥84	164 (73.5%)	59 (26,5%)	96 (84; 123)	0,368 (0,284; 0,476)	**<0,001**
Homozigotos HLA-A					
Não	966 (37.3%)	1,624 (62,7%)	41 (38; 43)	1,000	–
Sim	147 (46.4%)	170 (53,6%)	45 (35; 62)	0,872 (0,744; 1,021)	0,089
Homozigotos HLA-B					
Não	1,059 (38.2%)	1,713 (61,8%)	41 (38; 43)	1,000	–
Sim	54 (40.0%)	81 (60,0%)	51 (32; 69)	0,839 (0,671; 1,049)	0,124
Homozigotos HLA-DR					
Não	960 (36.2%)	1,689 (63,8%)	39 (39; 42)	1,000	–
Sim	153 (59.3%)	105 (40,7%)	73 (59; 104)	0,535 (0,439; 0,652)	**<0,001**

Abreviaturas – HR: razão de risco (calculada pelo modelo bruto de **Cox**); IC: intervalo de confiança; Q1: primeiro quartil; Q3: terceiro quartil; DP: desvio padrão. Notas – A idade refere-se à idade no momento do transplante renal para os receptores transplantados e à idade ao final do período do estudo para os candidatos que permaneceram ativos na lista de espera.

O grau de sensibilização e a homozigosidade HLA-DR foram identificados como os determinantes mais importantes para o aumento do tempo de espera na lista de transplantes. Pacientes com cPRA *≥*84 apresentaram 8% de chance de permanecer ativos na lista de espera. Da mesma forma, pacientes com cPRA <84 e homozigosidade HLA-DR também tiveram 8% de probabilidade de permanecer em lista. Em contraste, pacientes com cPRA <84 e sem homozigosidade do HLA-DR exibiram 84% de probabilidade de ter recebido um transplante. O modelo demonstrou acurácia de 66,36% (IC 95% 64,61%–68,07%), revelando poder discriminatório moderado. Portanto, um valor de corte de 84 para cPRA foi utilizado para análises subsequentes.

Os fatores associados a uma maior probabilidade de receber um transplante incluíram diabetes mellitus como doença de base (HR 1,177; p = 0,030) (Figura 1) e tipo sanguíneo A (HR 1,206; p < 0,001) ou AB (HR 1,419; p = 0,002), em comparação com o tipo sanguíneo O (Figura 2). Em contraste, uma menor probabilidade de transplante foi observada entre indivíduos mais velhos (HR 0,992; p < 0,001), aqueles com nível de cPRA *≥*84 (HR 0,368; p < 0,001) (Figura 3) e pacientes com homozigosidade HLA-DR (HR 0,535; p < 0,001) (Figura 4). Não foi encontrada associação signifi­cativa entre a homozigosidade HLA-A ou HLA-B e a probabilidade de transplante.

**Figura 1:** Curvas de *Kaplan-Meier* mostrando o tempo da inscrição na lista de espera até o transplante, estratificado pelo estado de diabetes mellitus.

**Figura 2:** Curvas de *Kaplan-Meier* mostrando o tempo da inscrição na lista de espera até o transplante, estratificado por tipo sanguíneo.

**Figura 3:** Curvas de *Kaplan-Meier* mostrando o tempo da inscrição na lista de espera até o transplante, estratificado por grau de sensibilização.

**Figura 4:** Curvas de *Kaplan-Meier* mostrando o tempo da inscrição na lista de espera até o transplante, estratificado pelo *status* homozigoto HLA-DR.

Na análise multivariada (Tabela 2), diversos fatores foram associados a uma maior probabilidade de receber um transplante renal. Esses fatores incluíram ter menos de 60 anos (HR 1,162; p = 0,009) e apresentar diabetes mellitus (HR 1,166; p = 0,046) e tipos sanguíneos A (HR 1,230; p < 0,001), AB (HR 1,447; p = 0,001) ou B (HR 1,162; p = 0,047), quando comparados ao tipo sanguíneo O. Um cPRA mais baixo (<84) (HR 2,718; p < 0,001) e a ausência de homozigosidade HLA-DR (HR 1,824; p < 0,001) também foram significativamente associados a uma maior probabilidade de transplante. De acordo com o modelo, o perfil com maior probabilidade prevista de receber um transplante (HR 9,72) foi o de uma mulher com menos de 60 anos, com diabetes, tipo sanguíneo AB, escore cPRA <84 e sem homozigosidade HLA-DR. Em contraste, o indivíduo com a menor probabilidade prevista (HR: 0,95) foi um homem com 60 anos ou mais, não diabético, com tipo sanguíneo O, escore cPRA *≥*84 e homozigosidade HLA-DR.

**Tabela 2:** Modelo de *Cox* ajustado para avaliação de fatores de risco para transplante

Características	HR	IC 95%	Valor de p
Sexo			
F	1,000	–	–
M	0,952	(0,865; 1,048)	0,314
Faixa etária			
<60 anos	1,162	(1,039; 1,300)	**0,009**
≥60 anos	1,000	–	–
Diabetes mellitus			
Não	1,000	–	–
Sim	1,166	(1,003; 1,356)	**0,046**
Tipo sanguíneo			
O	1,000	–	–
A	1,230	(1,110; 1,363)	**<0,001**
AB	1,447	(1,154; 1,815)	**0,001**
B	1,162	(1,002; 1,348)	**0,047**
Estado imunológico categorizado			
<84	2,718	(2,093; 3,529)	**<0,001**
≥84	1,000	–	–
Homozigotos HLA-DR			
Não	1,824	(1,497; 2,222)	**<0,001**
Sim	1,000	–	–

Abreviaturas – IC: intervalo de confiança.

Não se observou associação significativa entre a distribuição dos alelos HLA-A e HLA-B e o *status* de transplante. Entretanto, os alelos HLA-DR3 e DR8 foram menos frequentes entre os receptores (Tabela 3).

**Tabela 3:** Frequência dos alelos HLA-A, HLA-B e HLA-DR de acordo com o *status* do transplante

Características	Ativos na lista (n = 2.226)	Transplantados pelo sistema (n = 3.588)	Diferença (IC 95%)
HLA-A			
A1	164 (7,4%)	276 (7,7%)	-0,3% (-2,5%; 1,8%)
A2	542 (24,3%)	930 (25,9%)	-1,6% (-5,1%; 2,0%)
A3	192 (8,6%)	302 (8,4%)	0,2% (-2,1%; 2,5%)
A9	0 (-)	1 (0,03%)	-0,03% (-0,1%; 0,06%)
A11	110 (4,9%)	180 (5,0%)	0,1% (-1,9%; 1,7%)
A23	151 (6,8%)	245 (6,8%)	0,04% (-2,1%; 2,0%)
A24	156 (7,0%)	290 (8,1%)	-1,1% (-3,2%; 1,1%)
A25	19 (0,9%)	34 (0,9%)	-0,1% (-0,9%; 0,7%)
A26	67 (3,0%)	71 (2,0%)	1,0% (-0,3%; 2,3%)
A29	115 (5,2%)	166 (4,6%)	0,5% (-1,3%; 2,3%)
A30	193 (8,7%)	306 (8,5%)	0,1% (-2,2%; 2,5%)
A31	86 (3,9%)	106 (3,0%)	0,9% (-0,6%; 2,4%)
A32	65 (2,9%)	102 (2,8%)	0,1% (-1,3%; 1,5%)
A33	64 (2,9%)	134 (3,7%)	-0,9% (-2,3%; 0,6%)
A34	31 (1,4%)	56 (1,6%)	-0,2% (-1,2%; 0,8%)
A36	25 (1,1%)	30 (0,8%)	0,3% (-0,5%; 1,1%)
A43	1 (0,04%)	0 (-)	0,04% (-0,1%; 0,2%)
A66	32 (1,4%)	52 (1,4%)	0,01% (-1,0%; 1,0%)
A68	163 (7,3%)	206 (5,7%)	1,6% (-0,5%; 3,6%)
A69	4 (0,2%)	5 (0,1%)	0,04% (-0,3%; 0,4%)
A74	39 (1,8%)	85 (2,4%)	-0,6% (-1,8%; 0,5%)
A80	7 (0,3%)	11 (0,3%)	0,01% (-0,5%; 0,5%)
HLA-B			
B5	0 (-)	3 (0,1%)	-0,1% (-0,2%; 0,1%)
B7	151 (6,8%)	254 (7,1%)	-0,3% (-2,5%; 1,9%)
B8	102 (4,6%)	157 (4,4%)	0,2% (-1,6%; 2,0%)
B13	37 (1,7%)	40 (1,1%)	0,5% (-0,5%; 1,6%)
B14	9 (0,4%)	17 (0,5%)	-0,1% (-0,6%; 0,5%)
B15	28 (1,3%)	30 (0,8%)	0,4% (-0,5%; 1,3%)
B17	0 (-)	1 (0,03%)	-0,03% (-0,1%; 0,1%)
B18	111 (5,0%)	158 (4,4%)	0,6% (-1,3%; 2,5%)
B21	0 (-)	1 (0,03%)	-0,03% (-0,1%; 0,1%)
B27	62 (2,8%)	71 (2,0%)	0,8% (-0,6%; 2,2%)
B35	204 (9,2%)	375 (10,5%)	-1,3% (-3,9%; 1,3%)
B37	30 (1,3%)	32 (0,9%)	0,5% (-0,5%; 1,4%)
B38	25 (1,1%)	48 (1,3%)	-0,2% (-1,2%; 0,7%)
B39	67 (3,0%)	79 (2,2%)	0,8% (-0,6%; 2,2%)
B40	15 (0,7%)	9 (0,3%)	0,4% (-0,2%; 1,1%)
B41	26 (1,2%)	39 (1,1%)	0,1% (-0,9%; 1,0%)
B42	69 (3,1%)	92 (2,6%)	0,5% (-0,9%; 2,0%)
B44	214 (9,6%)	425 (11,8%)	-2,2% (-4,9%; 0,5%)
B45	69 (3,1%)	128 (3,6%)	-0,5% (-2,0%; 1,1%)
B46	1 (0,04%)	0 (-)	0,04% (-0,1%; 0,2%)
B47	4 (0,2%)	2 (0,1%)	0,1% (-0,2%; 0,4%)
B48	14 (0,6%)	11 (0,3%)	0,3% (-0,3%; 0,9%)
B49	72 (3,2%)	103 (2,9%)	0,4% (-1,2%; 1,9%)
B50	48 (2,2%)	67 (1,9%)	0,3% (-1,0%; 1,5%)
B51	152 (6,8%)	268 (7,5%)	-0,6% (-2,9%; 1,6%)
B52	51 (2,3%)	91 (2,5%)	-0,2% (-1,6%; 1,1%)
B53	111 (5,0%)	135 (3,8%)	1,2% (-0,6%; 3,1%)
B54	0 (-)	2 (0,1%)	-0,1% (-0,2%; 0,1%)
B55	21 (0,9%)	25 (0,7%)	0,2% (-0,6%; 1,1%)
B56	2 (0,1%)	9 (0,3%)	-0,2% (-0,5%; 0,2%)
B57	55 (2,5%)	110 (3,1%)	-0,6% (-2,0%; 0,8%)
B58	66 (3,0%)	115 (3,2%)	-0,2% (-1,8%; 1,3%)
B60	42 (1,9%)	63 (1,8%)	0,1% (-1,1%; 1,3%)
B61	37 (1,7%)	64 (1,8%)	-0,1% (-1,3%; 1,0%)
B62	69 (3,1%)	113 (3,1%)	-0,04% (-1,6%; 1,5%)
B63	27 (1,2%)	53 (1,5%)	-0,3% (-1,3%; 0,7%)
B64	19 (0,9%)	38 (1,1%)	-0,2% (-1,1%; 0,6%)
B65	83 (3,7%)	133 (3,7%)	0,02% (-1,7%; 1,7%)
B70	3 (0,1%)	9 (0,3%)	-0,1% (-0,5%; 0,3%)
B71	44 (2,0%)	74 (2,1%)	-0,1% (-1,3%; 1,2%)
B72	54 (2,4%)	106 (3,0%)	-0,5% (-1,9%; 0,9%)
B73	5 (0,2%)	2 (0,1%)	0,2% (-0,2%; 0,5%)
B75	9 (0,4%)	6 (0,2%)	0,2% (-0,3%; 0,7%)
B78	1 (0,04%)	5 (0,1%)	-0,1% (-0,3%; 0,2%)
B81	15 (0,7%)	22 (0,6%)	0,1% (-0,6%; 0,8%)
B82	2 (0,1%)	3 (0,1%)	0,01% (-0,3%; 0,3%)
HLA-DR			
DR1	232 (10,4%)	333 (9,3%)	1,1% (-1,2%; 3,5%)
DR3	29 (1,3%)	16 (0,4%)	**0,9% (0,1%; 1,6%)**
DR4	269 (12,1%)	422 (11,8%)	0,3% (-2,3%; 2,9%)
DR7	269 (12,1%)	453 (12,6%)	-0,5% (-3,1%; 2,1%)
DR8	170 (7,6%)	202 (5,6%)	**2,0% (0,1%; 4,1%)**
DR9	49 (2,2%)	57 (1,6%)	0,6% (-0,5%; 1,7%)
DR10	58 (2,6%)	82 (2,3%)	0,3% (-0,9%; 1,6%)
DR11	241 (10,8%)	440 (12,3%)	-1,4% (-4,0%; 1,1%)
DR12	37 (1,7%)	77 (2,1%)	-0,5% (-1,6%; 0,6%)
DR13	290 (13,0%)	512 (14,3%)	-1,2% (-4,0%; 1,5%)
DR14	62 (2,8%)	106 (3,0%)	-0,2% (-1,5%; 1,2%)
DR15	211 (9,5%)	371 (10,3%)	-0,9% (-3,2%; 1,5%)
DR16	63 (2,8%)	114 (3,2%)	-0,3% (-1,7%; 1,0%)
DR17	184 (8,3%)	334 (9,3%)	-1,0% (-3,3%; 1,2%)
DR18	62 (2,8%)	69 (1,9%)	0,9% (-0,4%; 2,1%)

Abreviaturas – IC: intervalo de confiança ajustado por *Goodman*.

## Discussão

Este estudo avaliou os fatores associados ao transplante renal com doador falecido e ao tempo de espera, em uma coorte regional de Minas Gerais, no período anterior à revisão nacional das diretrizes brasileiras de alocação de rins em setembro de 2025. Essa revisão incorporou a tipagem HLA ampliada e padronizou um sistema de pontuação de homozigosidade do receptor baseado nas identidades HLA-DRB1 e HLA-DQB1. Nossos achados destacam o papel central do *status* imunológico, particularmente da homozigosidade HLA e da sensibilização, bem como de fatores demográficos e clínicos, incluindo idade, diabetes mellitus e grupo sanguíneo, na determinação da probabilidade de transplante.

O diabetes mellitus associou-se a uma maior probabilidade de transplante renal nesta coorte. Esse achado deve ser interpretado no contexto da política brasileira de alocação, na qual o diabetes mellitus (tipo 1 ou tipo 2) constitui um critério clínico que confere pontos adicionais de prioridade. Consequentemente, candidatos diabéticos podem ser transplantados mais precocemente quando órgãos compatíveis se tornam disponíveis, enquanto aqueles que permanecem ativos na lista de espera representam uma coorte selecionada de sobreviventes com menor risco clínico imediato. O aumento da idade associou-se a uma menor probabilidade de transplante, provavelmente refletindo maior carga de comorbidades, piores desfechos esperados no pós-transplante e a alocação preferencial de rins de doadores falecidos para candidatos mais jovens. Como esperado, o grupo sanguíneo também influenciou o acesso, com os grupos A e AB apresentando maior probabilidade de transplante em comparação com o grupo sanguíneo O.

Nosso modelo preditivo demonstrou que a homo­zigosidade HLA-DR e o cPRA são determinantes-chave da redução do acesso ao transplante renal. A homozigosidade no *locus* HLA-DR associou-se a menor probabilidade de transplante, em consonância com estudos prévios que relatam tempos de espera prolongados entre candidatos homozigotos^15,16,17^. Receptores homozigotos podem ser desfavorecidos em sistemas de alocação que priorizam a compatibilidade HLA sem considerar adequadamente as restrições imunogenéticas impostas pela homozigosidade. Esse fenômeno foi previamente descrito por De Marco et al.^16^, que relataram uma frequência significativamente maior de homozigosidade HLA-DRB1 entre candidatos ao transplante em comparação com a população geral.

Pacientes hipersensibilizados, caracterizados por valores elevados de cPRA, enfrentam barreiras substanciais ao transplante devido à maior reatividade aloimune e à menor disponibilidade de doadores compatíveis^15,16,17,18,19^. Em nossa coorte, candidatos com cPRA *≥*84 apresentaram uma probabilidade marcadamente menor de transplante, reforçando o papel da sensibilização como um dos principais determinantes do tempo de espera prolongado. A interação entre a homozigosidade HLA e a sensibilização agrava ainda mais a desvantagem imunológica, uma vez que a homozigosidade pode contribuir para o desenvolvimento ou a persistência de níveis elevados de anticorpos anti-HLA.

Este estudo apresenta diversas limitações. Embora todos os pacientes elegíveis durante o período do estudo tenham sido incluídos, não foi realizado cálculo formal do tamanho amostral. O viés de seleção foi parcialmente mitigado ao se restringirem as análises a indivíduos avaliados por um único laboratório de referência; contudo, o delineamento retrospectivo introduz a possibilidade de confundimento residual e viés de informação. Além disso, os dados de comorbidades estavam ausentes ou incompletos em uma proporção substancial dos casos, ressaltando a necessidade de aprimorar a completude e a padronização das informações no registro do Sistema Nacional de Transplantes (SNT). A análise das frequências alélicas de HLA deve ser interpretada com cautela. Para lidar com o problema de comparações múltiplas, as proporções alélicas foram estimadas separadamente para cada grupo, utilizando intervalos de confiança simultâneos ajustados pelo método de *Goodman*, apropriado para distribuições multinomiais. Não foi realizado um teste formal de hipóteses entre múltiplos alelos e, portanto, a análise de frequência de HLA foi considerada exploratória. As diferenças observadas entre os grupos foram interpretadas de forma conservadora e consideradas relevantes apenas quando o IC da diferença não incluía zero.

## Conclusão

À medida que a carga global da DRC continua a aumentar, especialmente em países em desenvolvimento, a otimização das políticas de alocação de rins permanece essencial para melhorar a equidade e o acesso ao transplante. Este estudo demonstra que a homozigosidade HLA-DRB1 e a hipersensibilização estão associadas à redução do acesso ao transplante renal com doador falecido e a tempos de espera mais longos no Brasil.

Revisões recentes da política brasileira de alocação de rins, incluindo a classificação de pares doador-receptor como HLA-DRB1 idênticos quando ambos são homozigotos para o mesmo antígeno DRB1 e medidas voltadas aos candidatos sensibilizados, representam passos importantes para mitigar essas disparidades. A avaliação contínua e o aprimoramento dos algoritmos de alocação que considerem tanto a homozigosidade HLA quanto a sensibilização são necessários para melhorar a equidade e a eficiência do transplante renal no Brasil.

## Data Availability

Os dados gerados neste estudo podem ser disponibilizados mediante solicitação.
